# Temporal changes in reproductive success and optimal breeding decisions in a long-distance migratory bird

**DOI:** 10.1038/s41598-020-78565-y

**Published:** 2020-12-16

**Authors:** Cynthia Reséndiz-Infante, Gilles Gauthier

**Affiliations:** Département de Biologie, and Centre d’études Nordiques, Université Laval, 1045 Av. de la Médecine, Québec, QC G1V 0A6 Canada

**Keywords:** Population dynamics, Ecology, Phenology

## Abstract

Many avian migrants have not adjusted breeding phenology to climate warming resulting in negative consequences for their offspring. We studied seasonal changes in reproductive success of the greater snow goose (*Anser caerulescens atlantica*), a long-distance migrant. As the climate warms and plant phenology advances, the mismatch between the timing of gosling hatch and peak nutritive quality of plants will increase. We predicted that optimal laying date yielding highest reproductive success occurred earlier over time and that the seasonal decline in reproductive success increased. Over 25 years, reproductive success of early breeders increased by 42%, producing a steeper seasonal decline in reproductive success. The difference between the laying date producing highest reproductive success and the median laying date of the population increased, which suggests an increase in the selection pressure for that trait. Observed clutch size was lower than clutch size yielding the highest reproductive success for most laying dates. However, at the individual level, clutch size could still be optimal if the additional time required to acquire nutrients to lay extra eggs is compensated by a reduction in reproductive success due to a delayed laying date. Nonetheless, breeding phenology may not respond sufficiently to meet future environmental changes induced by warming temperatures.

## Introduction

Animals living in seasonal environments should optimize timing of breeding to maximize their reproductive success, which is typically highest when offspring are born during peak food availability^1–5^. Accordingly, the two most critical decisions in single-brooded breeding birds are probably when to start laying eggs (i.e. nest initiation date) and how many eggs to lay (clutch size), two decisions that are linked^6,7^. Birds can adjust both decisions to reach an optimal combination that yields maximal possible reproductive success. In long-distance migrants, weather encountered during migration and on the breeding grounds can have a strong influence on these decisions because it can affect body condition and feeding opportunities upon arrival^8,9^. Individual quality also plays a role in breeding decisions because high-quality individuals often arrive early at the breeding areas^10^. Early arriving birds are usually in better body condition, start nesting earlier, lay larger clutches and ultimately have a higher reproductive success than those arriving later^7^. In seasonal environments, delaying nest initiation due to a late arrival or poor body condition entails a cost in terms of the reproductive value of eggs as the chances of an egg producing a young reaching 1 year of age typically decrease over the egg-laying period in single-brooded species^6,7,11^. Nonetheless, breeding too early also entails potential costs. For instance, individuals laying very early may face more severe and unpredictable environmental conditions or higher egg predation risk due to reduced synchrony with the bulk of the population, which attenuates the predator-swamping effect occurring at high nesting densities^12,13^.

Climate warming may negatively affect the reproductive success of long-distance migrants because species of different trophic levels are likely to respond at different rates to climate warming^14^. Processes occurring at low trophic levels, such as onset of vegetation growth or insect outburst, typically advance at a faster rate in response to warming than those occurring at higher trophic level, such as the phenology of breeding birds^15^. This may result in mismatches between offspring hatch and peak food availability with negative consequences for offspring survival^14,16^. Phenotypic plasticity, which is the ability of individuals to change their response according to environmental variation, can allow migratory birds to cope with phenological changes induced by climate warming^17,18^. Birds can adjust laying date or clutch size to prevailing conditions, but often not enough to fully match the energetic needs of their offspring with phenological changes occurring at lower trophic levels^15,19,20^.

The consequences of trophic mismatch are exacerbated in Arctic-nesting geese because their breeding cycle is relatively long, they breed in highly seasonal environments where the summer is short and they are exposed to rapid climate warming^21^. In this environment, the time window to achieve optimal reproductive success is narrow, leaving few opportunities for individuals to adjust laying date to changing environmental conditions^22^. In greater snow geese (*Anser caerulescens atlantica*), early breeders have higher reproductive success than late breeders because they lay more eggs and their goslings hatch early, in synchrony with the peak in nitrogen concentration in their food plant^12, 23^. In contrast, late-hatched goslings face a trophic mismatch as they are exposed to food plants of decreasing nutritive quality^24^. Geese arriving late on the breeding ground or in poor body condition must delay laying to regain condition; however, a delay in egg laying reduces reproductive success due to late hatching. Therefore, females may trade off a reduction in clutch size for an advance in hatching date to reduce the fitness cost associated with a delayed laying^7^.

Prior research documented strong seasonal effects on several components of reproductive success in greater snow geese, from egg-laying until young reach 1 year of age^12^. Despite a pronounced warming trend on their breeding ground on Bylot Island (Canada), there was little change in laying date over the past 3 decades, thereby increasing the potential for a trophic mismatch^22,25^. We previously showed that seasonal patterns of reproductive success components followed different and sometimes opposite trends over 25 years in this population^25^. Clutch size decreased with laying date but this effect weakened over time. Success of nests initiated early and late in the season was lower than nests initiated near the population mean, and success increased over time. Finally, prefledging survival decreased with laying date but only near the end of the study period whereas postfledging survival consistently decreased in relation to laying date and over the study period. In this study, we integrated all these components, from egg laying until offspring reach 1 year of age, to estimate reproductive success and investigate how the relationship between success and laying data (i.e. seasonal change) varied over the study period. In a second analysis, we modelled seasonal changes in expected reproductive success over time for different clutch sizes laid at different dates. We hypothesised that, as the climate warmed, increasing trophic mismatch between the timing of gosling hatch and the timing of peak food quality^21,24^ over a 25-year period has reduced reproductive success of nests initiated late in the season. We also predicted that the optimal laying date yielding highest reproductive success occurred earlier over time in relation to the mean population laying date, and that optimal decisions with respect to laying date and clutch size changed due to individual adjustments.

## Results

### Seasonal pattern of reproductive success

**Figure 1:**
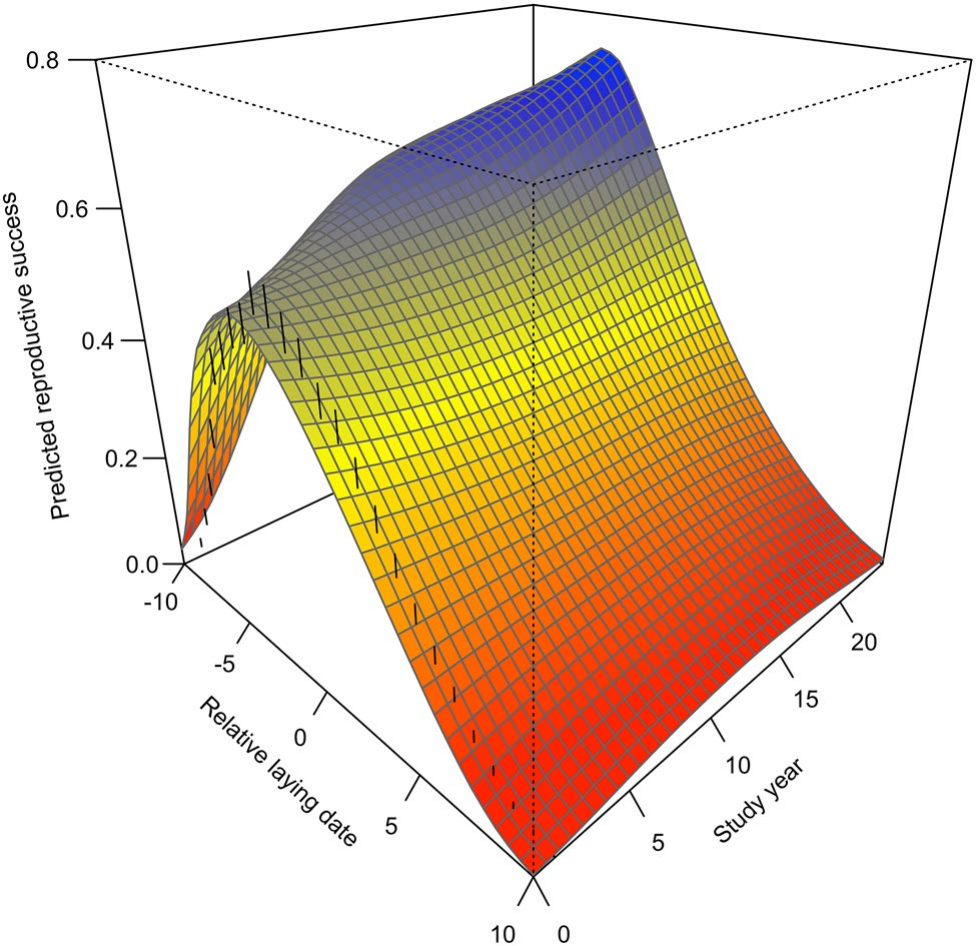
Predicted reproductive success of greater snow geese for each study year and relative laying date (from Day − 10 to + 10) from 1991 to 2015. Reproductive success is the number of offspring reaching 1 year of age. Day 0 is the annual median laying date of the population. Study year is a continuous variable, where 1991 is year 0. The surface represents the interpolation of reproductive success values for each relative day across study years. Blue indicates the highest values in the component, and red the lowest values. Confidence intervals at 95% (black vertical lines on left of surface) for each relative laying date are presented for year 0 (1991) and are similar across years. See also contour plot in Supplementary Fig. S1.

**Figure 2:**
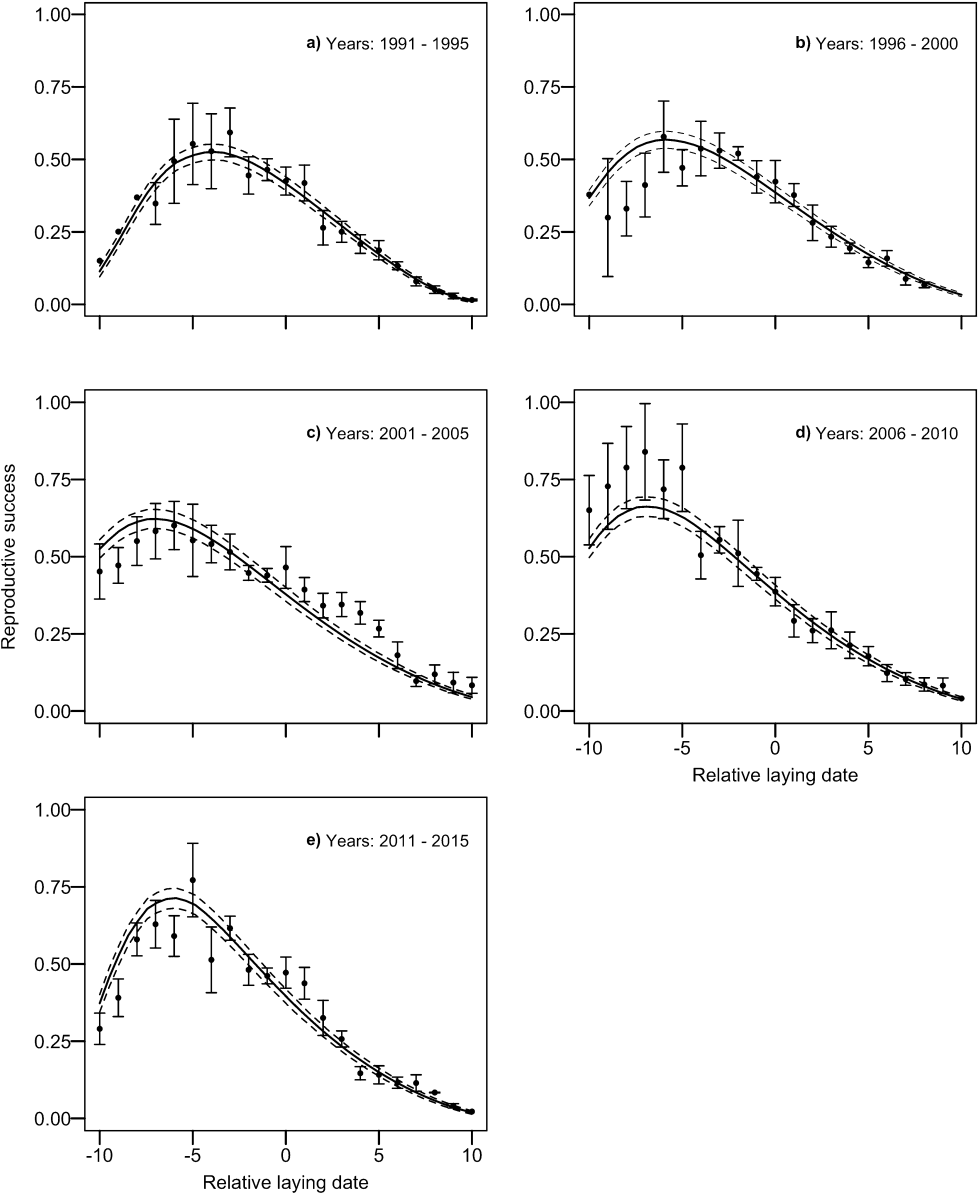
Observed reproductive success (black dots) in relation to relative laying date and predicted reproductive success (black line; black dotted lines represent the 95% CI) of greater snow geese derived from an analysis relating each reproductive component to laying date and study year (Table S1). Each data point is the mean over a 5-year period and the relationship is for the median year of the 5-year period. Error bars represent standard errors.

Predicted reproductive success showed large variations according to both laying date and study year (Fig. 1). Furthermore, across the study period, the predicted reproductive success derived from the seasonal and temporal analyses of individual components (Supplementary Table S1) tracked fairly well with the variations in observed reproductive success in relation to relative laying date (Fig. 2).

At the beginning of the study period, the earliest breeders (nests initiated at Day -10) had a low reproductive success of 0.03 young reaching 1 year of age. Success increased rapidly with laying date to peak at 0.52 young on Day -4 and declined steadily after that to < 0.01 young at Day + 10 (an average reduction of 0.036 young/day; Fig. 1; Supplementary Fig. S1). After 25 years, maximum reproductive success of early-nesting birds increased over time to reach 0.74 young, a 42% increase. This increase in maximum reproductive success resulted in a steeper seasonal decline after the date of peak success over the study period (Fig. 1). At the end of the study period, reproductive success declined from 0.74 young on Day − 6 to 0.01 young at Day + 10 (an average reduction of 0.046 young/day).

**Figure 3:**
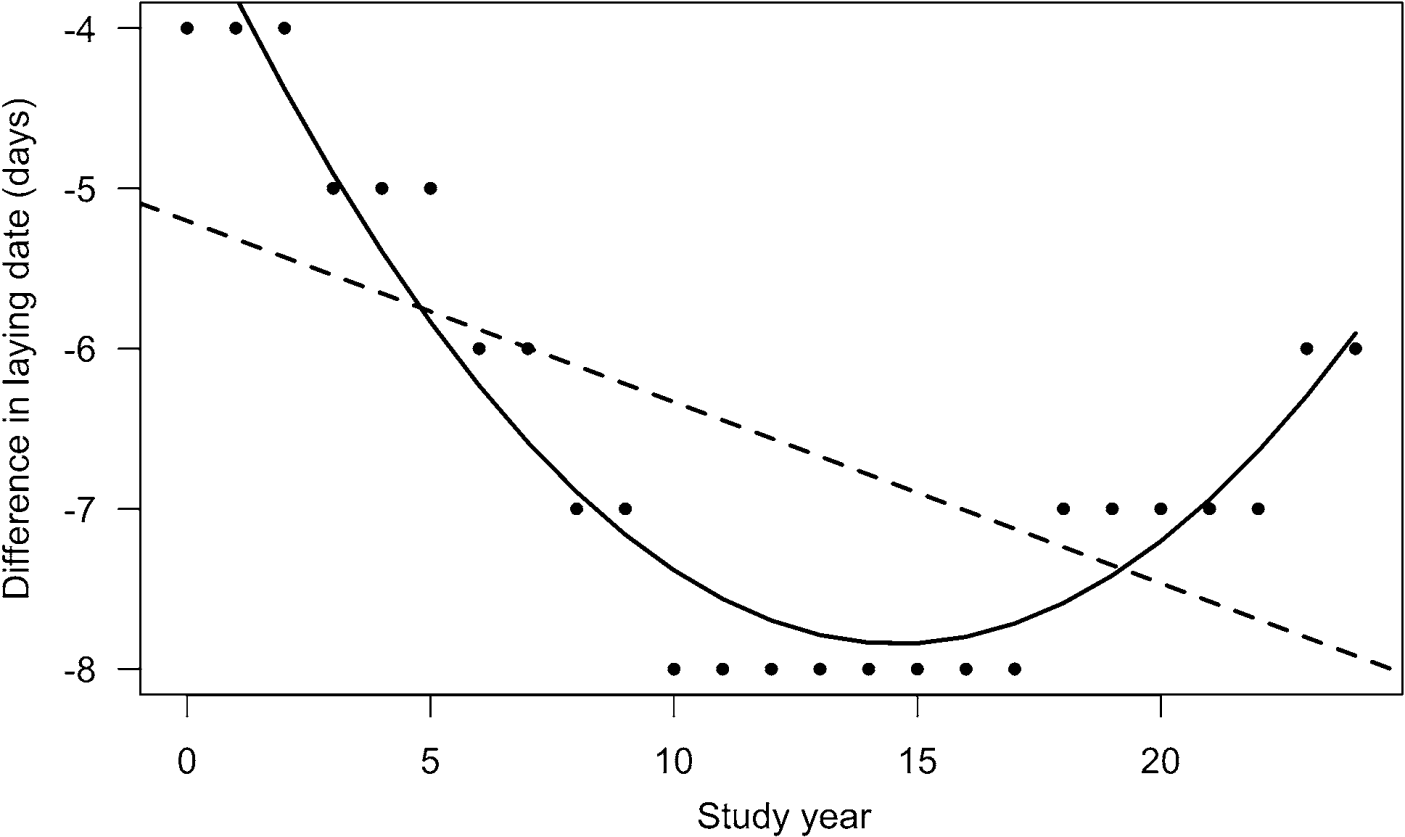
Difference between the laying date yielding the maximal reproductive success and annual median laying date of the greater snow goose population (Difference in laying date) from 1991 to 2015. Black dotted line shows the original linear model and black solid line the a posteriori quadratic model.

At the beginning of the study period, maximum reproductive success was achieved for birds laying on Day − 4 and gradually advanced to Day − 8 after 10 years. However, after 17 years, maximum success started to move back and was at Day − 6 at the end (Fig. 3). Overall, the difference between date of the maximum reproductive success and median laying date of the population increased over 25 years (slope = − 0.11, 95% CI − 0.18, − 0.05, R^2^ = 0.33). However, an a posteriori analysis where a squared term (year^2^) was added showed a better fit to the data than the linear model (slope year = − 0.64, 95% CI − 0.73, − 0.55; slope year^2^ = 0.022, 95% CI: 0.018, 0.025, R^2^ = 0.91).

### Clutch size, laying date and expected reproductive success

**Figure 4:**
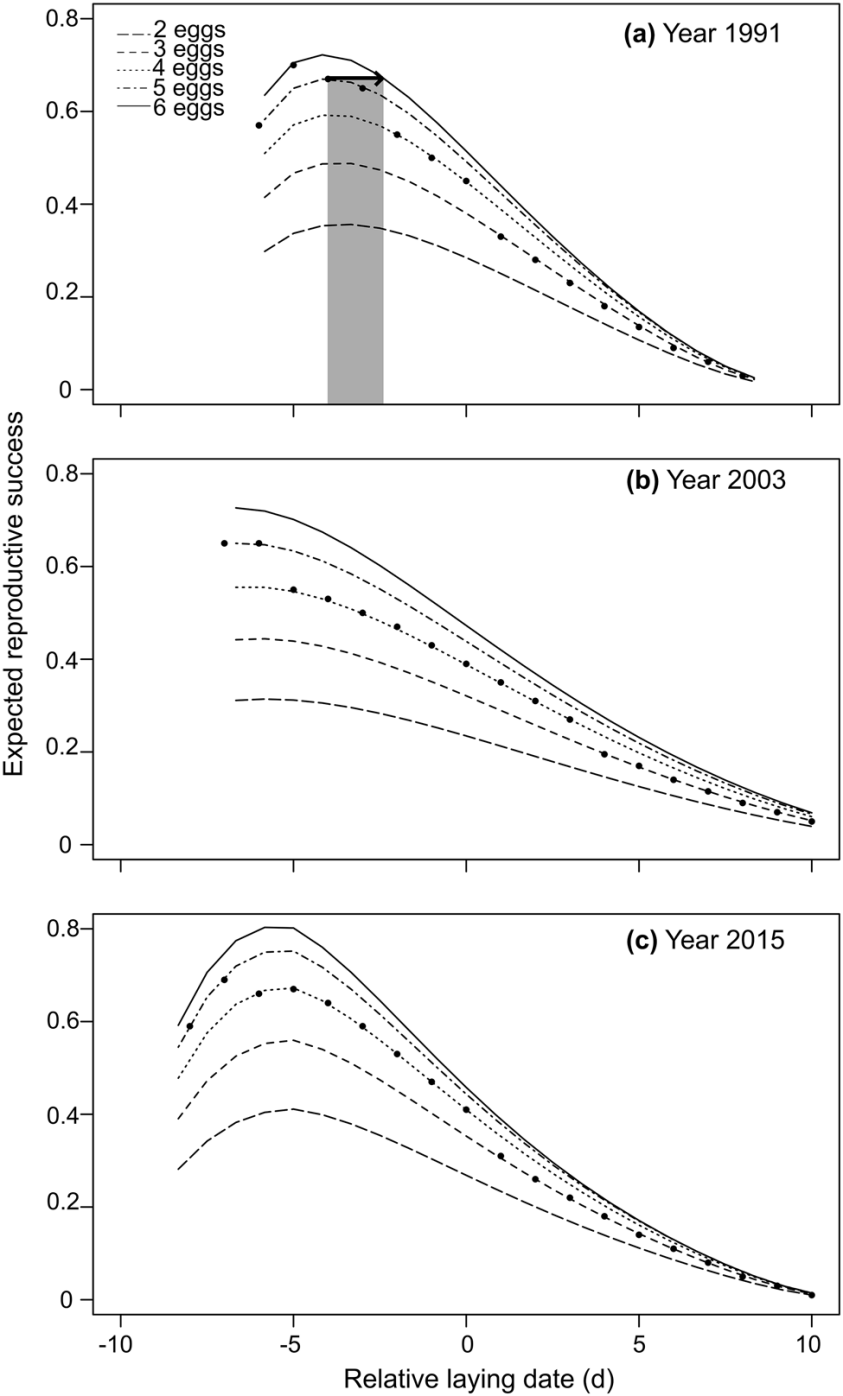
Expected reproductive success of greater snow geese in relation to relative laying date for birds laying a hypothetical clutch size of 2 to 6 eggs. Reproductive success is the number of offspring reaching 1 year of age. Figures show expected reproductive success at the beginning (**a**), half-way (**b**) and at the end (**c**) of the study period. Dots represent mean observed clutch size for each laying dates in those years. On panel (**a**), the black arrow and associated grey shading represents the maximum number of days (here, 1.6 days) that a bird about to lay 5 eggs on Day − 4 could delay laying to acquire enough nutrient to lay an extra egg and achieve a higher reproductive success (see text for details).

The expected reproductive success of birds laying a hypothetical clutch size of 2 to 7 eggs (Supplementary Figs. S2 and S3) showed seasonal and annual patterns of variation similar to the predicted reproductive success (Fig. 1). Although we calculated expected reproductive success from Day − 7 to + 10 for most study years, it should be noted that some combinations of clutch size and laying data were not observed. For instance, clutches of 6 and 7 are virtually absent after Day + 4 and the same applies for clutches of 2 before Day − 5 (Supplementary Fig. S4). On the same graph, we superimposed the seasonal variation in expected reproductive success of birds laying a clutch size of 2 to 6 eggs at the beginning, halfway and at the end of the study period (Fig. 4). The difference in expected reproductive success among various clutch sizes decreased in larger clutches, and seasonal decline in success was steeper in larger clutches than in smaller ones. Consequently, all lines tended to converge for birds laying on Day + 5 or later, especially at the beginning and the end of the study period. For almost all laying dates, the observed clutch size was lower than our model’s clutch size yielding the highest reproductive success. For early laying birds (Day − 7 to − 3 in 1991, − 7 to − 6 in 2003 and − 8 to − 7 in 2015), observed clutch size was about one egg less than the clutch size yielding the highest success (Fig. 4). However, for progressively later laying dates, this difference was two eggs and even sometimes three eggs.

## Discussion

The general pattern of seasonal variation in reproductive success of greater snow geese was maintained over a 25-year period, with the highest success achieved for birds laying before the population median. The maximum reproductive success increased over time and the date at which it was achieved advanced by at least 2 days although the median egg-laying date did not change in the population^25^. Consequently, the seasonal decline in reproductive success became steeper over time. Our analysis also suggests that the clutch size laid by geese was lower than the clutch size yielding the maximum reproductive success for most laying dates throughout the study period.

Even though egg-laying is fairly synchronized in greater snow geese (87% of the nests are initiated over 11 days on average), reproductive success shows strong seasonal variations. Reproductive success was highest in early-nesting birds, mainly from Day − 6 to − 4, suggesting a clear advantage for birds to lay early. However, laying too early also entails a cost, as reproductive success of the earliest breeders was low. The poor success of the earliest nests is driven mostly by the nesting success component^25^. These nests suffer higher predation by arctic foxes (*Vulpes lagopus*), the main cause of failure^26^, than nests initiated near the population mean. Unlike the latter group, the earliest nests do not benefit from the predator-swamping effect provided by high goose densities^27,28^.

Reproductive success of birds laying before the population median, which includes those with the highest success, showed a temporal increase. This could be partly explained by the warming trend documented in the area over the past three decades^22^. As previously documented in this population, climate can have both direct and especially indirect effects on goose reproduction^29,30^. Warming has increased plant production and advanced plant phenology at our study site^22,24^. Better feeding conditions in spring may have enhanced body condition of early nesting birds, thereby improving nest attendance of incubating females and reducing predation risk^31^, which likely contributed to a higher reproductive success^30^. After hatch, food quality is an important determinant of gosling growth as they require young plants with a high nitrogen content^23,24^. Feeding conditions of early-hatched goslings may have improved due to warmer summers because they still hatch close to the peak in nutritive quality of plants. Therefore, the temporal increase in reproductive success of early-nesting geese could also result from an increase in prefledging survival of goslings^25^. Improved reproductive success up to fledging in response to seasonal warmness has also been reported in a long-term study of sub-arctic lesser snow geese (*Anser caerulescens caerulescens*)^32^.

Reproductive success of birds laying at the population median or after changed little in contrast to those laying earlier. This is somewhat surprising considering that prefledging survival of late-hatched goslings tended to decrease over time^25^, possibly because the mismatch between hatching date and the peak in plant nutritive quality increased^21,24^. Recent evidence shows that black brant goslings (*Branta bernicla nigricans*) can respond to decreasing food availability with behavioural adjustments^33^ (reduced resting periods and increased search time for food), which could partly buffer the negative effects of trophic mismatch. In some colonies, habitat destruction due to overabundant snow goose populations has reduced gosling survival and reproductive success^21,34^. However, at our study site, colony size has remained constant and below the carrying capacity of the habitat^35^, and we have no evidence of a reduction in plant availability for geese over the study period^22,36^. The general increase in nesting success over time in our population may have also partly offset the negative effect of reduced prefledging survival on the overall reproductive success of late-nesting birds.

The laying date that achieved highest reproductive success advanced over time in the first half of the study period but levelled off in the second half. As previously found in an earlier study^12^, birds laying on Day − 4 had the highest reproductive success at the beginning of the study period, but in recent years this occurred on Day -6. This suggests strong selection for birds to lay earlier. Climate warming has disrupted trophic interactions in seasonal environments and has increased selection for early breeding in several wild populations^17,19,37^. Our results suggest that this also applies to our population, probably because warming has pushed the peak plant nutritive quality for goslings earlier in the season, thereby advancing the date when maximum reproductive success is achieved. Despite this apparent temporal increase in selection pressure for early laying, average laying date did not advance in our population^22^. Interestingly, a finer analysis revealed that laying date of earliest breeders advanced by 2 days, suggesting a possible adjustment for some components of the population, perhaps the highest quality individuals^25^. An a posteriori analysis suggested that the difference between the laying date yielding the highest reproductive success and the median laying date of the population may have been reduced in the last few years of the study period. We do not have an explanation for this possible reversal, as climate continued to warm locally in recent years and we have no evidence that other environmental factors could be involved.

The absence of a general response of the population to an apparent increase in selection pressure for early laying may be due to other phenological constraints such as the arrival date of birds on the breeding ground^20^. Departure of geese from wintering grounds is largely driven by photoperiod, a fixed environmental cue, although movement through successive stopovers may be influenced by timing of food availability^38^. A slower rate of warming at lower latitudes may prevent geese from adjusting their migration schedule and arrival time to conditions prevailing on their Arctic breeding ground. Considering that birds need to recover body condition for egg formation after their arrival^39^, this may impose a minimum delay between arrival and laying, limiting the ability of most individuals to advance their laying date^40^. Our results suggest that warming may have created a selection pressure that favours early breeders in this population due to a trophic mismatch^24^ and could have contributed indirectly to increased success by reducing vulnerability of nests to predation. The opposite is true for late breeders, which may explain why the seasonal decline in breeding success became steeper in our population over time.

The strong decline in reproductive success in seasonal environments can be explained by the condition-dependent optimisation model^6^, which predicts the optimal combination of clutch size and laying date in relation to arrival time and body condition of individuals. This model is based on a trade-off between clutch size and laying date as a strategy to maximise individual fitness. In this model, time becomes a major constraint limiting clutch size, as previously found in tree swallows (*Tachycineta bicolor*)^41^. An analysis completed in the earlier years of our study period found that the observed clutch size matched the clutch size that yielded maximal reproductive success for most laying dates in greater snow geese^12^, in accordance with the condition-dependent optimisation model^6^. Females were apparently trading off an additional egg for earlier laying to achieve the maximum possible reproductive success, thereby leading to a strong seasonal decline in clutch size at the population level.

Our analysis, which uses the same approach but over a much longer time period (25 years instead of 7 years^12^), found that observed clutch size was lower than the one yielding maximal reproductive success for various laying dates. However, such an analysis overlooks an important aspect of the condition-dependent optimisation model^6^, which is the time required to acquire enough nutrients to lay an additional egg. In greater snow geese, nutrients invested in egg-production come from a combination of body reserves accumulated during migration and from feeding at arrival on breeding ground, during prelaying^39^. For a bird to lay an additional egg to increase its reproductive success (Fig. 4), it will need time to accumulate enough nutrients to form the additional egg. Therefore, it is possible that the time required to acquire those nutrients could cause a delay in the start of egg-laying resulting in a greater reduction in reproductive success than the success gained by laying the extra egg. For instance, at the beginning of the study period, a female having sufficient nutrient reserves to lay 5 eggs on Day − 4 (i.e. observed mean clutch size for that date) would need time to acquire more nutrients to produce an extra egg, which would delay laying. If the feeding time required to acquire these nutrients is ≥ 2 days, then the reproductive success associated with this 6-egg clutch will actually be lower than the one expected by laying 5 eggs on Day − 4 (Fig. 4a). In this example, laying a smaller clutch size at an earlier date (i.e. 5 eggs on Day − 4) could still be the optimal solution for that individual in terms of reproductive success. Therefore, females arriving on the breeding ground may face a conflict between laying as early as possible to avoid a mismatch between hatching date of their offspring and peak nutritive quality in plants or delaying laying to gain additional nutrients to form extra eggs^7^. The solution to this conflict would depend on female individual body condition at arrival and the rate of nutrient gain, information that was not available in our study.

In this study, we were unable to monitor the same individuals from egg-laying to young reaching 1 year of age. Therefore, we had to combine independent samples collected at various breeding stages (Supplementary Fig. S5) to obtain the overall reproductive success^25^ which may reduce some of the individual variability. For the same reason, we do not have information on some factors known to influence reproductive success at the individual level such as female age^10,42^, which limits our ability to conduct genetic analysis at the individual level. Our analysis is also based on single-season rather than lifetime reproductive success. Snow geese are known to show variation and trade-off in their individual reproductive decisions between years^43,44^ and thus it is likely that much of the variability found in laying date and clutch size reflect individual decisions. Nonetheless, we cannot exclude that some of the variation observed could be due to change in the population structure over time rather than individual adjustments. Finally, geese may skip breeding in some years^43^ and thus our dataset, which is based only on individuals that actually attempted to breed, may not be a totally random sample of the population. Including the decision of breeding or not breeding could somewhat affect the seasonal variation in reproductive success^44^.

## Conclusion

Long-distance arctic migrants like geese are under a strong pressure to lay early in the season to maximise their reproductive success. Our analysis suggests that over a 25-year period, reproductive success of early breeders increased and the laying date that maximises success became progressively earlier as climate warmed. However, mean laying date of the population did not advance. This is possibly due to constraints encountered during migration, which resulted in an apparent increase in the selection pressure for that trait. Nonetheless, as predicted by the condition-dependent optimisation model^6^, our results may still be consistent with the hypothesis that geese are maximising their reproductive success at the individual level by trading off additional eggs in their clutch for an earlier laying date, but this would depend on the time required to acquire nutrients to lay extra eggs.

## Methods

### Study species and study area

The greater snow goose overwinters on the east coast of the United States and migrates to breeding grounds in the eastern Canadian High-Arctic, with a major stopover in southern Quebec^45^. Though it is a mixed capital/income breeder, egg production depends largely on Arctic food resources available before and during laying^38^. At the individual level, clutch size and laying date are highly variable in response to environmental conditions and also vary with age as clutch size increases and laying date advances when individuals become older^42,46^. A single clutch is laid per year, and predation is the main cause of nesting failure^26,28^. Young and adults are strictly herbivorous, feeding predominantly on leaves, grasses and sedges.

We studied the snow goose population of the south plain of Bylot Island, Nunavut, Canada (72° 53.49′ N, 79° 54.38′ W) where ca. 20,000 pairs breed^47^. Typical landforms on the south plain include low hills with gentle slopes and large flat areas. Mesic tundra dominates the landscape, but wetlands associated with ponds and tundra polygons are very common^35^. Most geese nest in a main colony located in the central portion of the south plain, but some individuals also nest in a dispersed fashion across the area^48^.

### Field methods

Goose reproduction has been monitored annually on Bylot Island since 1989. In this study, we used data collected during the full reproductive season (June to August) from 1991 to 2015. Intensive nest searches were conducted throughout egg-laying and early incubation periods to ensure that both early and late nests were found. We used two main sampling schemes throughout the study period. The first one consisted of systematic nest searches within a single main plot (ca. 50 ha) located in the center of the breeding colony. The second sampling scheme consisted of systematic nests searches in smaller plots (1 ha to 4 ha) randomly located throughout the goose colony. The number of plots was variable each year, as we aimed to monitor ~ 100 nests in this scheme. Finally, some nest opportunistically found (15% of all nests) were also monitored and used in the analysis to maximize sample size. Reproductive parameters were found to be consistent among these sampling schemes^25^. We monitored 283 nests annually on average although this number varied among years (range: 130–493 nests) because the annual reproductive effort at the population level varied considerably in response to prevailing environmental conditions^29^. Nests were monitored, and visited at least twice, until hatch. Within 24-h after hatch, we marked goslings with web-tags before they left the nest. Monitored nests that could not be visited at hatch time were visited later to record the presence of membranes as an indicator of a successful hatching event. Right before fledging, we captured family groups (parents with their young) in mass banding drives and each bird received a metal leg-band^49^. Recaptures of leg-banded adults in previous years and web-tagged goslings at hatch were recorded. We also obtained recovery data from bands reported by hunters to the Bird Banding Laboratory of the U.S. Geological Service. All applicable institutional and/or national Canadian guidelines for the care and use of animals were followed and the field protocols were approved by the Animal Care Committee of Université Laval.

### Reproductive success components

Because geese are precocial birds and use different areas through the breeding cycle, we could not follow the same individuals from the egg-laying stage until 1 year of age. Therefore, data used for estimating reproductive components comes from three different samples: monitored nests, goslings web-tagged at hatch and birds banded near fledging (Supplementary Fig. S5).

Laying date was the date on which the first egg was laid in a nest. We back-calculated laying date using three different methods. For nests found during the laying period, we back-calculated laying date assuming that 1 egg was laid every 33 h^50^. For nests found during incubation and with a known hatching date, we back-calculated laying date from hatching date based on its clutch size and assuming a 23-day incubation period starting at the last-laid egg. For nests found during incubation but with unknown hatching date, we used individual egg density (determined from egg measurements and mass) to estimate incubation stage and to back-calculate laying date. We defined hatching date of a brood as the date on which at least half of the clutch hatched. To adjust for inter-annual environmental variability, we centred individual values on the annual median laying or hatching dates of the population (i.e. relative day with respect to the annual population median set equal to 0). Hereafter, centred dates are referred to as relative laying and hatching dates.

We decomposed reproduction into several successive components from egg-laying until young reach 1 year of age (Supplementary Fig. S5)^25^. Total clutch laid (TCL) was the maximum number of eggs found in a nest after the start of incubation. We excluded observations of nests where TCL was 1 egg, most likely a consequence of partial predation, and > 7 eggs, considered a result of intraspecific brood parasitism^12^. Nesting success (NS) was the probability of at least one egg hatching in a nest. Egg survival (ES) was the proportion of eggs surviving to hatch time in successful nests and was calculated as ES = CSH/TCL, where CSH = clutch size at hatch. Hatching success (HS) was the proportion of eggs that hatch in a successful nest and was calculated as HS = GLN/CSH, where GLN = number of goslings leaving a nest.

We estimated prefledging survival (S1) from goslings web-tagged at hatch that survived over the brood-rearing period and were recaptured at banding time. We could not use conventional capture-recapture methods here because we had a single recapture event. S1 was thus estimated for individual broods where at least one gosling was recaptured as N_recaptured_/N_marked_. However, this approach overestimates survival because broods in which all young die (total brood loss) cannot be detected even if their parents are recaptured because parents are not marked. We corrected S1 estimates for total brood loss as described in Supplementary Methods. Finally, postfledging survival (S2) was the probability of a juvenile surviving from fledging until young reach 1 year of age and was estimated by applying standard capture-recapture methods to the dataset of banded birds (see details in^25^).

### Data analyses

#### Estimated reproductive success

We estimated reproductive success (RS), defined as the number of young reaching 1 year of age per reproductive female, by the product of all individual components defined above. We calculated reproductive success for each relative laying date (*d*) of the season (i.e. laying dates from Day − 10 to + 10) and each study year using the following equation^12,42^.

1 $$RS_{d} = \, TCL_{d} \times NS_{d} \times ES_{d} \times HS_{d} \times S1_{{d^{\prime}}} \times S2_{{d^{\prime}}}$$

We used Eq. (1) in two ways. First, we estimated reproductive success in each year and laying date based on our original dataset (hereafter referred to as observed reproductive success). Second, we used predicted values derived from an analysis relating each reproductive component to laying date and study year as reported in^25^ (hereafter referred to as predicted reproductive success). Confidence intervals (95%) for the predicted reproductive success were computed using 10,000 Monte Carlo simulations. In each simulation, we randomly sampled a value from the distribution of predicted values for each reproductive component to obtain the sampling variance used to calculate confidence intervals.

Because posthatch components S1 and S2 were analysed using relative hatching dates, we adjusted the hatching date to its corresponding laying date (d′) to match the response variable used in the prehatch components. Because each egg is laid at ~ 33 h interval, a clutch size larger than the mean will delay hatching by 1 day for each additional egg laid, and conversely hatching will be advanced by 1 day for each egg removed. Therefore, the modal clutch size of 4 eggs was subtracted from the observed clutch size for each day of the season (TCL_d_) and this value added to d to estimate the hatching date d′ corresponding to laying date d (Eq. 2) as in^12^.

2 $${d}^{^{\prime}}= d+({\stackrel{-}{TCL}}_{d}-4)$$

To assess how well the predicted reproductive success tracked the observed reproductive success, we proceeded as follows. We divided the study period into five 5-year periods and calculated the mean observed reproductive success for each relative laying date within each 5-year period. We then plotted these values over the relationship between predicted reproductive success and relative laying date for the median year of each 5-year period.

#### Expected reproductive success

We evaluated the consequences of individual breeding decisions (laying date and clutch size) on reproductive success according to our model. Expected reproductive success was calculated for a bird laying a given clutch size over the range of 2 to 7 eggs on relative dates ranging from − 10 to + 10 across the 25-year study period. We estimated the expected offspring survival at the nest (OS; Eq. 3), which is the probability of producing a gosling leaving the nest, for each day of the season and year with the following equation:

3 $$E\left( {OS} \right)_{d} = NS_{d} \times ES_{d} \times HS_{d}$$

Expected reproductive success for different hypothetical clutch size *C* (from 2 to 7 eggs; Eq. 4) and laying date of the season (from − 10 to + 10) were calculated as the product of expected offspring survival at the nest (OS) and posthatch components:

4 $$E\left( {RS} \right)_{d} = C \times OS_{d} \times S1_{{d^{\prime\prime}}} \times S2_{{d^{\prime\prime}}}$$

For the same reason as for the calculation of observed reproductive success, relative laying date of posthatch components was adjusted (d") when combined with prehatch components depending on the value of *C* using the following equation.

5 $$d^{\prime\prime} \, = \, d \, + \, \left( {C \, {-} \, 4} \right)$$

## Supplementary Information

Supplementary Information

## Data Availability

The datasets used for this study are available in: Gauthier, G., & Cadieux, M.-C. Monitoring of greater snow goose reproduction on Bylot Island, Nunavut, Canada, v. 1.1 (1989–2019). Nordicana, D41. https://doi.org/10.5885/45570CE-2D00DCA728074FA7 (2020).
