# Supplementary material for: Temporal changes in reproductive success and optimal breeding decisions in a long-distance migratory bird
Source: Sci Rep. 2020 Dec 16;10:22067. doi: 10.1038/s41598-020-78565-y (PMC7744573; doi:10.1038/s41598-020-78565-y)
Supplement: Supplementary file 1 — Supplementary Information [file 41598_2020_78565_MOESM1_ESM.pdf]

Supplementary information

Temporal changes in reproductive success and optimal breeding decisions in a long-distance migratory bird

Cynthia Reséndiz-Infante[1]*, Gilles Gauthier[1]

[1] Département de biologie, and Centre d'études nordiques, Université Laval, 1045 Av. de la Médecine, Québec, QC, Canada G1V 0A6

*Corresponding author: cynthia.resendiz-infante.1@ulaval.ca

Supplementary Table S1. Parameter estimates with 95% confidence intervals relating various components of reproductive success to study year (year) and laying or hatching dates. Laying and hatching date refer to relative dates estimated as deviations from annual median values. Relative laying date was used to analyze prehatching components (total clutch laid, nesting success, egg survival, hatching success), whereas relative hatching date to analyze posthatch components (prefledging and postfledging survival). Details of these analyses are presented in [1].

Reproductive success component	Parameter	Estimate	Low CI	High CI
**Total clutch laid (TCL)**	Intercept	3.81	3.78	3.85
	laying date	-1.33E-01	-1.43E-01	-1.22E-01
	year	-4.72E-03	-8.22E-03	-1.21E-03
	$year^2$	2.52E-04	-2.82E-04	7.86E-04
	laying date * year	1.47E-03	2.59E-04	2.69E-03
	laying date * $year^2$	-4.66E-04	-6.42E-04	-2.90E-04
**Nesting success**	Intercept	4.67	4.54	4.81
	laying $date^2$	-7.84E-03	-1.06E-02	-4.96E-03
	year	3.69E-02	2.90E-02	4.49E-02
	$year^2$	2.32E-03	1.03E-03	3.62E-03
	laying $date^2$ * $year^2$	-1.02E-04	-1.51E-04	-5.16E-05
	nest age	2.68E-02	1.79E-02	3.57E-02
**Egg survival**	Intercept	2.20	2.13	2.27
	laying date	-2.96E-02	-5.74E-02	-1.74E-03
	year	-9.56E-03	-1.93E-02	2.11E-04
**Hatching success**	Intercept	2.58	2.45	2.72
	year	5.75E-02	4.05E-02	7.51E-02
	$year^2$	7.63E-03	5.09E-03	1.03E-02
**Prefledging survival**	Intercept	0.33	0.24	0.41
	hatching date	3.16E-02	-6.73E-03	6.99E-02
	$year^2$	-5.87E-05	-1.51E-03	1.40E-03
	hatching date * $year^2$	-9.07E-04	-1.54E-03	-2.76E-04
**Postfledging survival**	Intercept	-0.66	-0.78	-0.54
	hatching date	-0.15	-0.20	-0.10
	year	-0.16	-0.30	-0.01

Supplementary Figure S1. Contour plot showing the predicted reproductive success of greater snow geese as a function of relative laying date and study year. Reproductive success is the number of offspring reaching 1 year of age. Contour interval = 0.1. See Fig. 1 in main text for additional information.

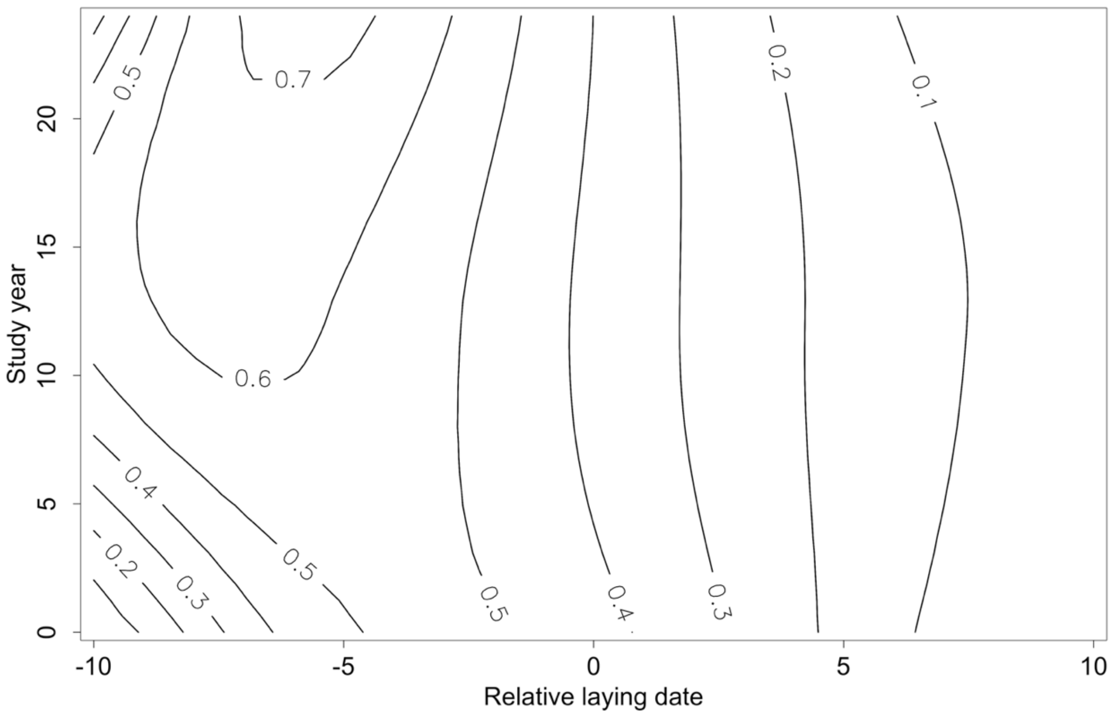

Supplementary Figure S2. Expected reproductive success of greater snow geese at different hypothetical clutch size (2 to 7 eggs, from (a) to (f) respectively) for each study year and relative laying date (from Day -10 to +10) from 1991 to 2015. Reproductive success is the number of offspring reaching 1 year of age. Study year is presented as a continuous variable, where 1991 is year = 0. The surface represents the interpolation of reproductive success values for each relative day across the study years. Blue indicates the highest values in the component, and red the lowest values. Black bars represent 95% confidence intervals of year 0 (1991). See also contour plots in Figure S3.

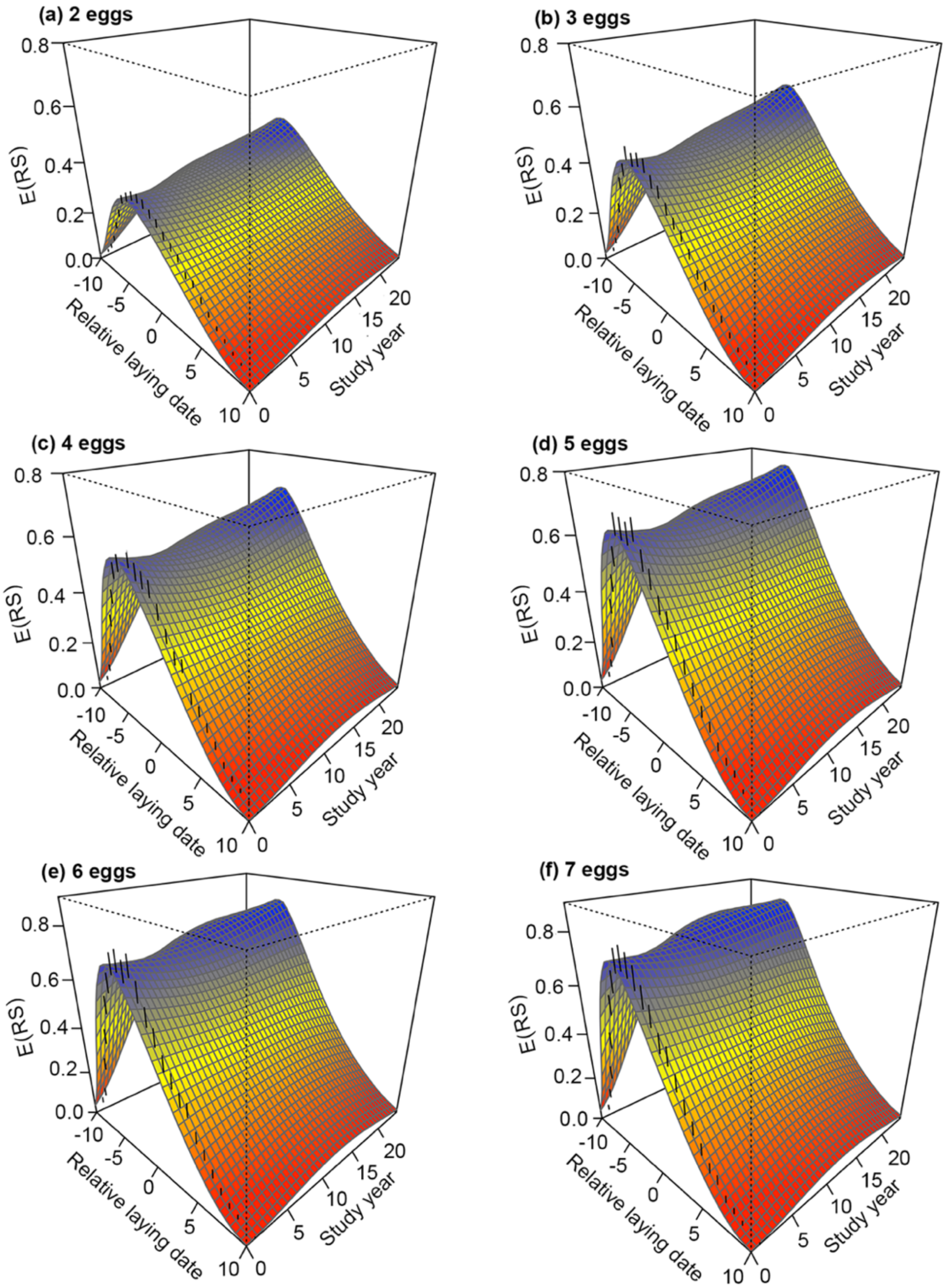

Supplementary Figure S3. Contour plots showing the expected reproductive success of greater snow geese at different hypothetical clutch size of 2–7 eggs, from (a) to (f) respectively, as a function of study year and relative laying date. Reproductive success is the number of offspring reaching 1 year of age. Contour line interval = 0.05. See Fig. S2 for additional information.

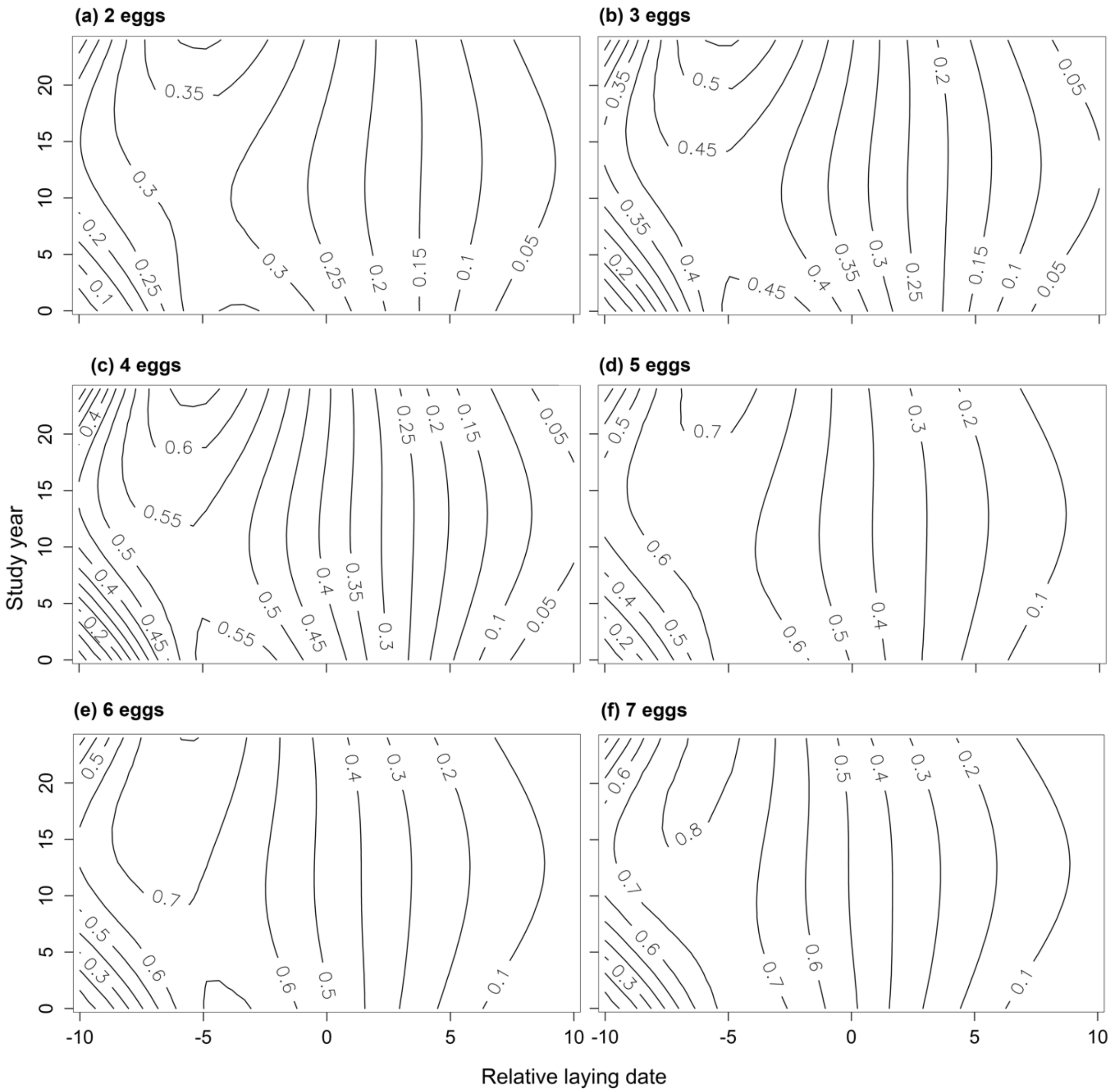

Supplementary Figure S4. Frequency distribution of observed clutch size of greater snow geese from 2 to 7 eggs according to relative laying date. Data from 1991-2015.

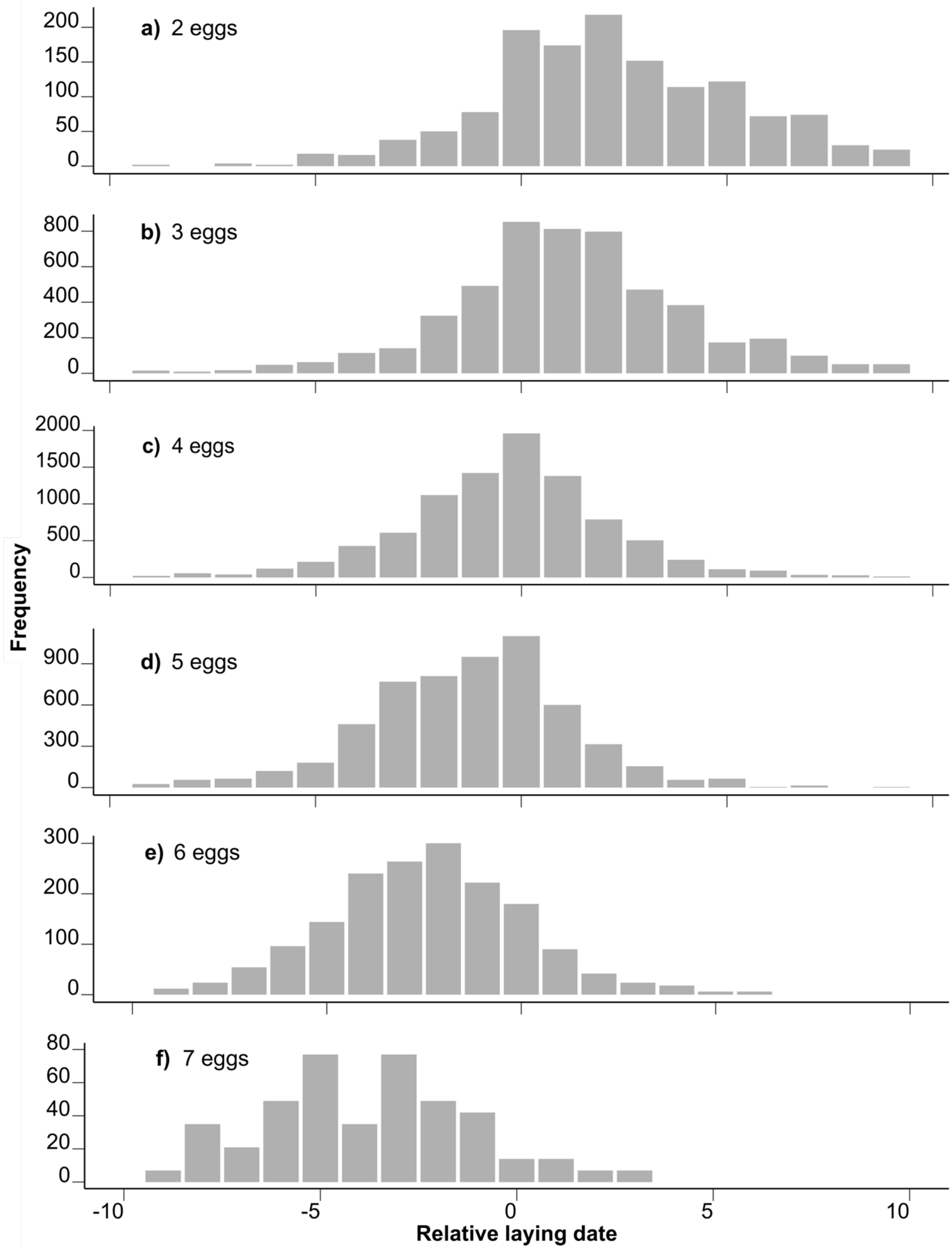

Supplementary Figure S5. Reproductive success components of greater snow geese from egg-laying until birds reach 1-year of age. The lines under the figure refer to the three datasets used to estimate the reproductive components (monitored nests, web-tagged goslings and banded birds). NS = nesting success, ES = egg survival, HS = hatching success, S1 = prefledging survival, S2 = postfledging survival (from [1]).

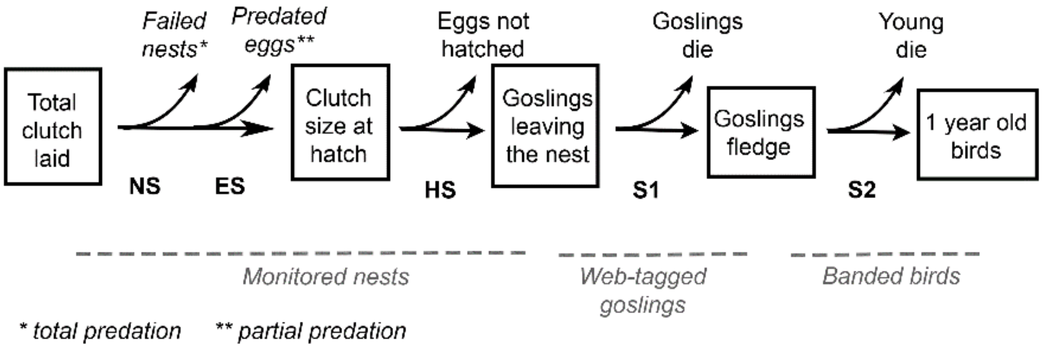

Supplementary Methods. Estimation of total brood loss

We estimated goslings dying in broods where all young disappear, i.e. total brood loss (TBL) [2]. We calculated TBL for each year using the ratio number of goslings (N_G):number of adult (N_A) among birds captured at banding and the average brood size (BS) at that time. We determined average brood size in free-ranging birds using spotting scopes just before the banding period on >100 broods annually. We used the formula:

$$TBL = 1 - \frac{N_G/N_A}{BS/2}$$

This estimation assumes that all young were accompanied by both parents at banding and that only successful breeders (i.e. those that hatched goslings) were caught. These assumptions could be met in our data because young are attended by both parents over the summer and brood-mixing and adoption are uncommon in snow geese[3]; thus, when young survive, parents and young are caught together. Moreover, only parents that hatched young could be captured because most non-breeders and unsuccessful nesters have completed their moult and have regained flight capabilities when goose banding occurs in August[4]. TBL varied among years (range: 0.01–0.41) and averaged 0.18 but showed no significant trend over time (slope = -4.42E-03, 95% CI: -1.13E-02, 2.43E-03); thus values were averaged across years. To correct prefledging survival estimates (S1) determined by the proportion of web-tagged goslings recaptured in surviving broods, we multiplied this value by the proportion of broods where at least one young survive, which was given by 1-$\overline{TBL}$.

References

1. Reséndiz-Infante, C., Gauthier, G. & Souchay, G. Consequences of a changing environment on the breeding phenology and reproductive success components in a long-distance migratory bird. *Pop. Ecol.* **62,** 284–296 (2020).
2. Gauthier, G. & Brault, S. Population model of the greater snow goose: projected impacts of reduction in survival on population growth rate. 65-80. BDJ Batt (Ed). *The greater snow goose: report of the Arctic Goose Habitat Working Group.* Arctic Goose Joint Venture Special Publication. U.S. Fish and Wildlife Service, Washington D.C. and Canadian Wildlife Service (1998).
3. Williams, T. D., Cooch, E. G., Jefferies, R. L. & Cooke, F. Environmental degradation, food limitation and reproductive output: juvenile survival in lesser snow geese. *J. Anim. Ecol.* **62,** 766–777 (1993).
4. Reed, E. T., Bêty, J., Mainguy, J., Gauthier, G. & Giroux J.-F. Molt migration in relation to breeding success in greater snow geese. *Arctic*, **56,** 76–81 (2003).
